# The effect of unilateral chest drainage for transpulmonary pressure during mechanical ventilation

**DOI:** 10.1186/s40981-023-00664-2

**Published:** 2023-10-28

**Authors:** Takayuki Hasegawa, Yuzo Iseki, Atsuyuki Hosono, Satoki Inoue

**Affiliations:** Division of Anesthesia and Pain Medicine, Fukushima Medical University Hospital, 1 Hikarigaoka, Fukushima City, Fukushima, Fukushima 960-1295 Japan

**Keywords:** Chest drainage, Chest tube, Mandatory mechanical ventilation, Transpulmonary pressure

## Abstract

**Introduction:**

Chest tube drainage is usually performed through an underwater seal at a level of 10–20 cmH_2_O. Based on the definition of transpulmonary pressure, continuous chest drainage creates continuous negative pressure, decreasing pleural surface pressure and increasing transpulmonary pressure. We investigated how unilateral chest drainage could affect the tidal volume or driving pressure during mandatory mechanical ventilation.

**Methods:**

This study was an experimental study using a lung-thoracic model and anesthesia ventilator. Tidal volume was set to 300 mL with pressure-controlled ventilation or volume-controlled ventilation. Left tidal volume and right tidal volume were measured independently using respirometers with positive end-expiratory pressure (PEEP) levels of 0, 10, and 20 cmH_2_O. Simultaneously, left negative pressure of the chest drainage was changed to 0, 10, and 20 cmH_2_O.

**Results:**

In all conditions, a tidal volume of 300 mL was achieved. In both pressure-controlled ventilation and volume-controlled ventilation, the left tidal volume increased with the application of chest drainage at 10 cmH_2_O when the PEEP level was 0 cmH_2_O, but left tidal volume decreased with the application of chest drainage at 20 cmH_2_O. Furthermore, when PEEP was 10 cmH_2_O, the left tidal volume decreased in proportion to the pressure of thoracic drainage. The right tidal volumes changed inversely with their counterpart left tidal volumes.

**Conclusion:**

Unilateral chest drainage caused unbalanced ventilation of the left and right lungs regardless of pressure-controlled ventilation or volume-controlled ventilation.

## Introduction

Placement of a chest tube is one of the most common medical procedures. Placement of a chest tube is considered for pneumothorax, malignant pleural effusion, empyema, complicated parapneumonic pleural effusion, traumatic hemothorax, infectious pleural effusion or post thoracic surgery [1]. If chest drainage is required, it can be performed through an underwater seal at a level of 10–20 cmH_2_O [1].

Transpulmonary pressure (TPP) is defined as the pressure difference between the airway opening and the pleural surface [2, 3]. Based on this definition, continuous chest drainage creates a continuous negative pressure, decreases pleural surface pressure, and increases TPP. The chest tube is often placed in a unilateral thoracic cavity. Therefore, unilateral thoracic pleural surface pressure is reduced by unilateral chest drainage, which may result in unbalanced ventilation of the left and right lungs. Matsumoto et al. reported the effects of chest drainage on tidal volumes during pressure-controlled ventilation (PCV) in an experimental study [4], although they were unable to verify the effects of chest drainage on volume-controlled ventilation (VCV). Furthermore, the aforementioned study assumed a situation in which a chest tube was inserted into one thorax and one lung, and did not assume a situation in which a chest tube was inserted unilaterally into either the left or right thoracic cavity under two pairs of thoraxes and lungs.

In the current study, we investigated how unilateral chest drainage could affect ventilation conditions, while PCV and VCV are being conducted during mandatory mechanical ventilation. In this experimental study, we used a model consisting of two sets of lung and thoracic cavity ventilated by an anesthesia ventilator.

## Materials and methods

This study is an experimental study using an anesthesia ventilator and handmade lung-thoracic cavity model; it is not a human trial. Therefore, informed consent and ethics committee approval were waived.

**Fig. 1:**
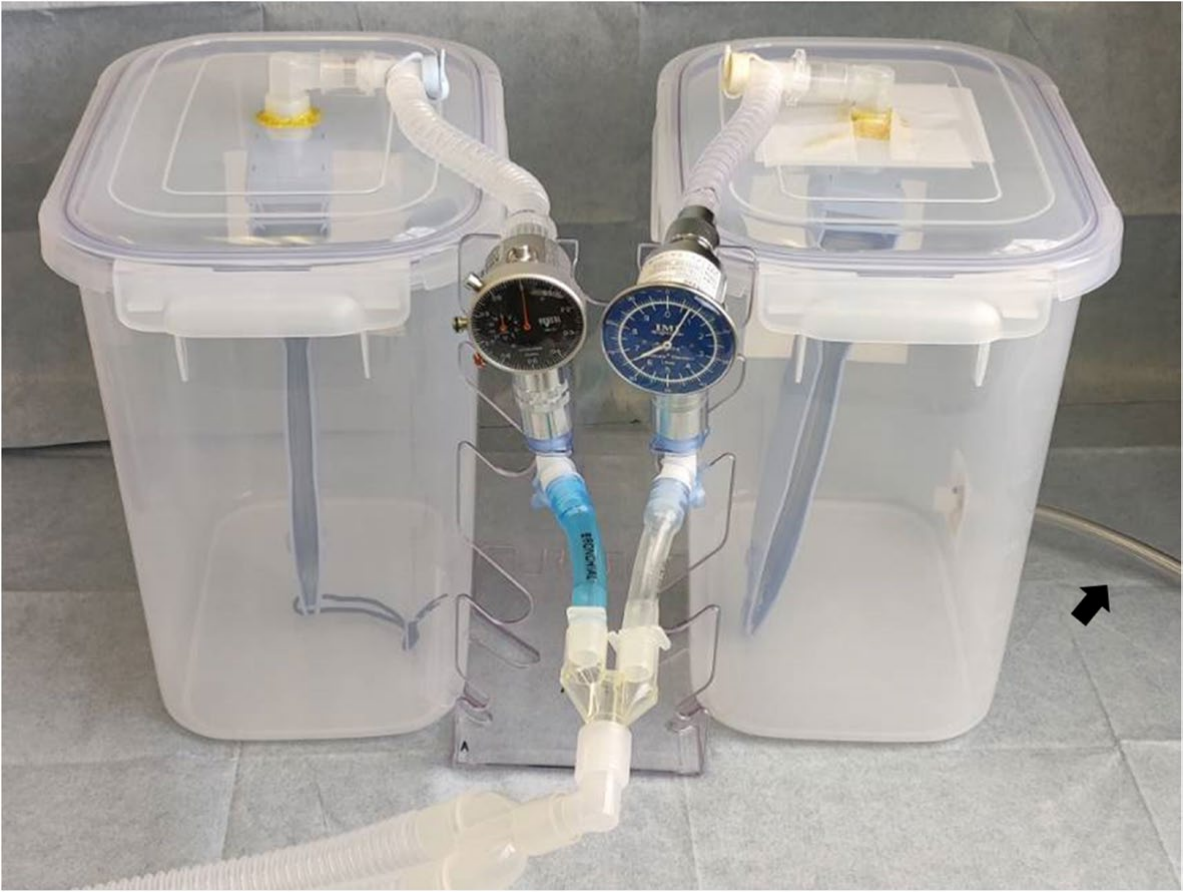
A lung-thorax cavity model, to which respirometers and a chest tube were connected, is shown. Two plastic containers each contain a 1-L test lung, which can be ventilated via the standard elbow connector. The tidal volume of each test lung was measured by a flow sensor connected to the ventilator side of the standard elbow connector, and the total tidal volume of both was measured by the flow sensor built into the ventilator. The plastic container has a 1.5-m tube connected to the thorax model (arrow), which is connected to the chest tube drainage system

We prepared a handmade lung-thoracic cavity model using a 1-L test lung and a plastic food storage container (Fig. 1). An airtight plastic food storage container (22 cm × 33.3 cm × 30.5 cm) was used to simulate a thoracic cavity. We made a hole in the lid of the container and connected a standard elbow connector (15-mm inner diameter/22-mm outer diameter) with strong adhesive gel to prevent air leakage. We connected the test lung (Venti.Plus™, GaleMed Corporation, Taiwan) and the standard elbow connector inside the container and considered the test lung and the container as the lung and the thoracic cavity, respectively. Two of these sets were prepared. We took each lung-thoracic cavity model, made a small hole on the side of the container, and connected a 1.5-m flexible tube to the hole with strong adhesive gel. We called it “the left lung-thoracic cavity model” for the convenience of the experiment. We connected the container and the chest drainage system (MERA continuous suction unit MS-009, Senko Medical Instrument Mfg. Co., Ltd. Tokyo) through the flexible tube. We simulated the lungs of a ventilated patient with a chest tube inserted into the left thoracic cavity through the lung-thoracic cavity model and the chest drainage system. GE Datex Ohmeda Aestiva 5 (GE Healthcare Japan, Tokyo) was used as the anesthesia ventilator.

### Experimental protocol

In the present study, the anesthesia ventilator setting with respiratory rate of 10 breaths per minute and inspiratory to expiratory ratio of 1:2 was not changed. Left tidal volume (LTV) and right tidal volume (RTV) were measured independently by respirometers; the LTV was measured by Datex-Ohmeda RM 121 (GE Healthcare Japan, Tokyo), and the RTV was measured by Haloscale Standard™ (KoKo PFT, LLC, CO, USA). We confirmed in advance that these two respirometers worked equally well, although we could not avoid using different brands of respirometers for the sake of resources. Plateau pressure (Ppl) and total tidal volumes (TTVs) derived from the right and left lungs were measured with a GE/Datex Ohmeda Flow Sensor (GE Healthcare Japan, Tokyo) mounted in the Aestiva 5. Prior to measurement, we ran targeted ventilation settings for 2 min to stabilize the parameters. These parameters were measured 10 times consecutively.

First, the ventilation mode was set to PCV. Ventilation was started with the following setting: positive end-expiratory pressure (PEEP) level at 0 cmH_2_O and PCV pressure to achieve TV 300 mL. Under this ventilator setting, we varied the negative pressure of the left chest drainage from 0, 10, and 20 cmH_2_O and measured the LTVs, the RTVs, and the Ppls (setting 1). Second, the ventilator setting was changed as follows: PEEP level at 10 cmH_2_O and PCV pressure to achieve TV 300 mL. Under this ventilator setting, we varied the negative pressure of the left chest drainage from 0, 10, and 20 cmH_2_O and measured the LTVs, the RTVs, and the Ppls (setting 2). Third, VCV was applied with a squared wave and a pause time of 30% of the inspiratory time. Ventilation was restarted with the following setting: 0 cmH_2_O of PEEP and 300 mL of TV. We then varied the negative pressure of the left chest drainage from 0, 10, and 20 cmH_2_O and measured the LTVs, the RTVs, and the Ppls (setting 3). Finally, the ventilator setting was changed as follows: 10 cmH_2_O of PEEP and 300 mL of TV. We varied the negative pressure of the left chest drainage from 0, 10, and 20 cmH_2_O and measured the LTVs, the RTVs, and the Ppls (setting 4). Theoretical end-expiratory TPP (EE-TPP), peak transpulmonary pressure (peak-TPP), and driving pressure were calculated from the Ppls and PEEPs as well as the negative pressures generated by chest drainage. These are expressed as “EE-TPP = PEEP—negative pressure generated by chest drainage,” “peak TPP = Ppl—negative pressure generated by chest drainage,” and “driving pressure = peak TPP—EE-TPP.”

### Statistical analysis

All measurements are reported as mean and standard deviation (SD). Means and SDs are presented in figures and tables. Sample size calculation and statistical comparisons were not performed for the following reasons. First, this study was an observational exploratory research study. Second, the variations in the sample values were very small and not clinically meaningful variations; however, these considerably small variations increased statistical power enough to allow them to be judged as statistically significantly different. We determined that it was not appropriate to interpret the study results from inferential statistical significance. Therefore, sample size calculations or statistical comparisons were not performed.

## Results

**Fig. 2:**
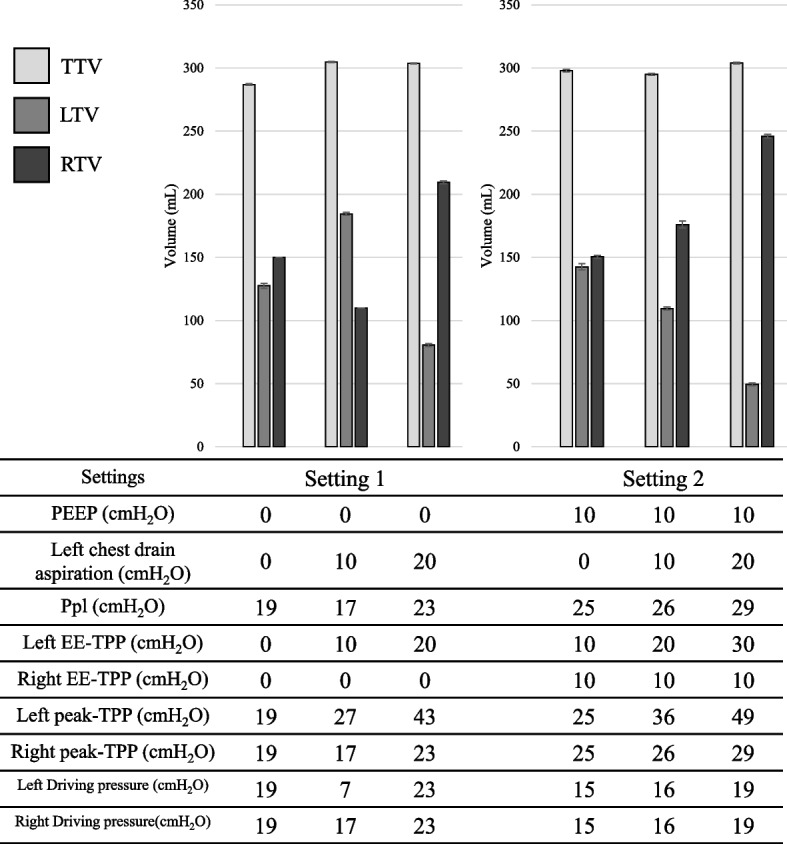
Experimental results for settings 1 and 2 are shown as bar graphs, with TTV, LTV, and RTV. PEEP and left chest drainage are also shown for each setting. The measured Ppl is shown, and the calculated EE-TPP at the left and right end of expiration is shown as left or right EE-TPP. *TTV*, total tidal volume; *RTV*, right tidal volume; *LTV*, left tidal volume; *PEEP*, positive end-expiratory pressure; *Ppl*, plateau pressure; *EE-TPP*, end-expiratory transpulmonary pressure; *TPP* transpulmonary pressure

**Fig. 3:**
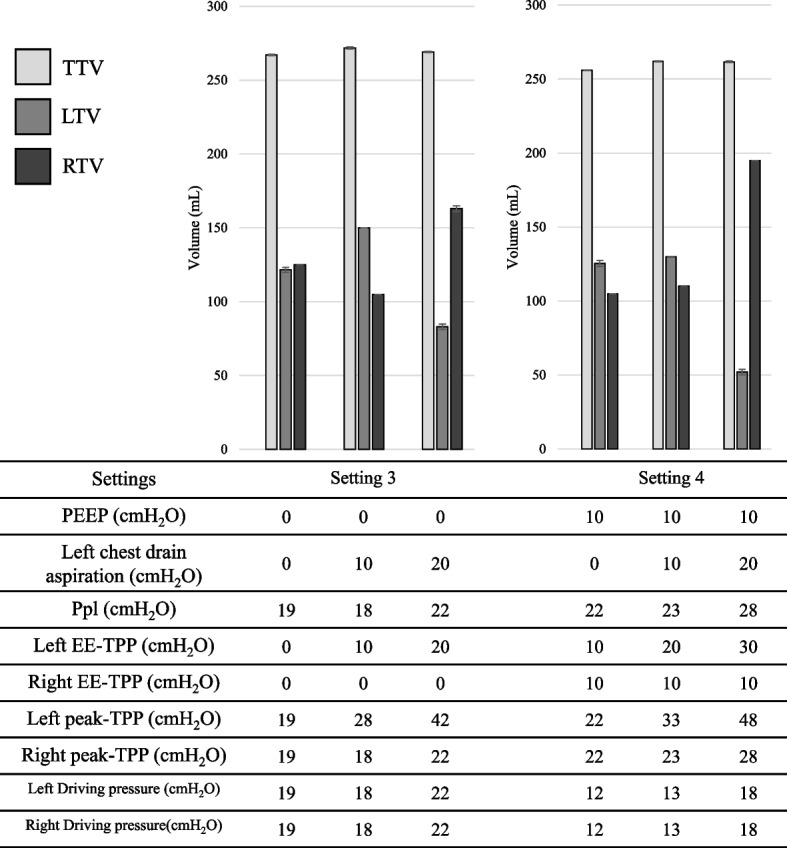
Experimental results for settings 3 and 4 are shown as bar graphs, with TTV, LTV, and RTV. PEEP and left chest drainage are also shown for each setting. The measured Ppl is shown, and the calculated EE-TPP at the left and right end of expiration is shown as left or right EE-TPP. *TTV*, total tidal volume; *RTV*, right tidal volume; *LTV*, left tidal volume; *PEEP*, positive end-expiratory pressure; *Ppl*, plateau pressure; *EE-TPP*, end-expiratory transpulmonary pressure; *TPP*, transpulmonary pressure

The LTVs, the RTVs, the TTVs, and the Ppls in settings 1–4 are shown in Table 1. In addition, the bar graphs for the TVs were constructed so that the results could be clearly seen (Figs. 2 and 3). The values of the EE-TPPs, the peak-TPPs, and driving pressures were also included in the tables in Figs. 2 and 3.

**Table 1:** LTVs, the RTVs, the TTVs, and the Ppls in settings 1–4

Settings	PEEP (cmH_2_O)	Left chest drainage (cmH_2_O)	TTV (mL) ± SD	LTV (mL) ± SD	RTV (mL) ± SD	Ppl (cmH_2_O) ± SD
Setting 1	0	0	287 ± 0.9	128 ± 2.5	150 ± 0.0	19 ± 0
	0	10	305 ± 0.6	185 ± 1.5	110 ± 0.0	17 ± 0
	0	20	304 ± 0.7	81 ± 1.5	210 ± 1.5	23 ± 0
Setting 2	10	0	298 ± 1.5	142 ± 3.4	151 ± 1.5	25 ± 0
	10	10	295 ± 1.0	110 ± 1.5	176 ± 3.7	26 ± 0
	10	20	304 ± 0.9	50 ± 1.5	246 ± 2.0	29 ± 0
Setting 3	0	0	264 ± 0.5	122 ± 2.3	125 ± 0.0	19 ± 0
	0	10	272 ± 0.9	150 ± 0.0	105 ± 0.0	18 ± 0
	0	20	269 ± 0.5	83 ± 2.4	163 ± 2.4	22 ± 0
Setting 4	10	0	256 ± 0.3	125 ± 2.7	105 ± 0.0	22 ± 0
	10	10	262 ± 0.4	130 ± 0	110 ± 0.0	23 ± 0
	10	20	262 ± 0.6	52 ± 2.4	195 ± 0.0	28 ± 0

Positive end-expiratory pressure (PEEP) of the ventilator, left side chest tube drain aspiration pressure, total tidal volume (TTV), left tidal volume (LTV), right tidal volume (RTV), and plateau pressure (Ppl) for each setting are shown. PEEP and left chest tube drainage are equal to the set values; TTV, LTV, RTV, and Ppl are the mean and standard deviation of the measurements. In all settings, the target value for tidal volume was 300 mL. Pressure-controlled ventilation was used in settings 1 and 2, and volume-controlled ventilation was used in settings 3 and 4

For the right lung, the PEEP was equal to the EE-TPP. Therefore, the right EE-TPPs were 0 cmH_2_O during setting 1, 10 cmH_2_O during setting 2, 0 cmH_2_O during setting 3, and 10 cmH_2_O during setting 4. For the left lung, the absolute value of applied chest drainage added to the PEEP was equivalent to the EE-TPP. Therefore, the left EE-TPPs were greater than their right EE-TTPs by the absolute values of chest drainage.

The Ppls to achieve 300 mL of TVs showed either a decrease or no significant changes with the application of 10 cmH_2_O of chest drainage in both PCV and VCV. This tendency was more often observed when no PEEP was applied. However, applying 20 cmH_2_O of chest drainage increased the Ppls at all ventilation settings. Based on the definitions of the EE-TPP and the peak-TPP, these values changed in parallel with the Ppls. Overall, driving pressures also changed in parallel with the Ppls; however, applying the PEEP decreased driving pressures.

In terms of the LTVs, application of 10 cmH_2_O of chest drainage increased the LTVs without the PEEP regardless of PCV or VCV. However, application of 20 cmH_2_O of chest drainage decreased the LTVs. When 10 cmH_2_O of PEEP was applied, the LTVs were decreased almost proportionally to the chest drainage. Because the experimental settings were made to achieve 300 ml of TVs, the RTVs changed inversely to the counterpart the LTVs.

## Discussion

In the current observational exploratory research study, we demonstrated that unilateral chest drainage can cause unbalanced ventilation of the left and right lungs regardless of PCV or VCV, which means that the TTVs provided by a ventilator may not be delivered equally to both lungs by the negative pressure created by unilateral chest drainage. In addition, it was demonstrated that the way TVs were delivered to each lung can depend on the EE-TPP and peak-TPP of each lung.

In the present study, the left lung compliance improved and then deteriorated compared to the right lung depending on the degree of chest drainage and the PEEP. Finally, the Ppls drastically increased with the PEEP combined with strong chest drainage. This phenomenon can be explained by the characteristics of the pressure–volume (P–V) curve of the test lung used in this study. The P–V curve of the lung has a lower inflection point (LIP) and an upper inflection point (UIP). It is known that lung compliance increases drastically when the inspiratory pressure passes near the LIP, and it decreases, and overdistention of the lung occurs causing harmful alveolar stretching when the inspiratory pressure passes above the UIP [5].

**Fig. 4:**
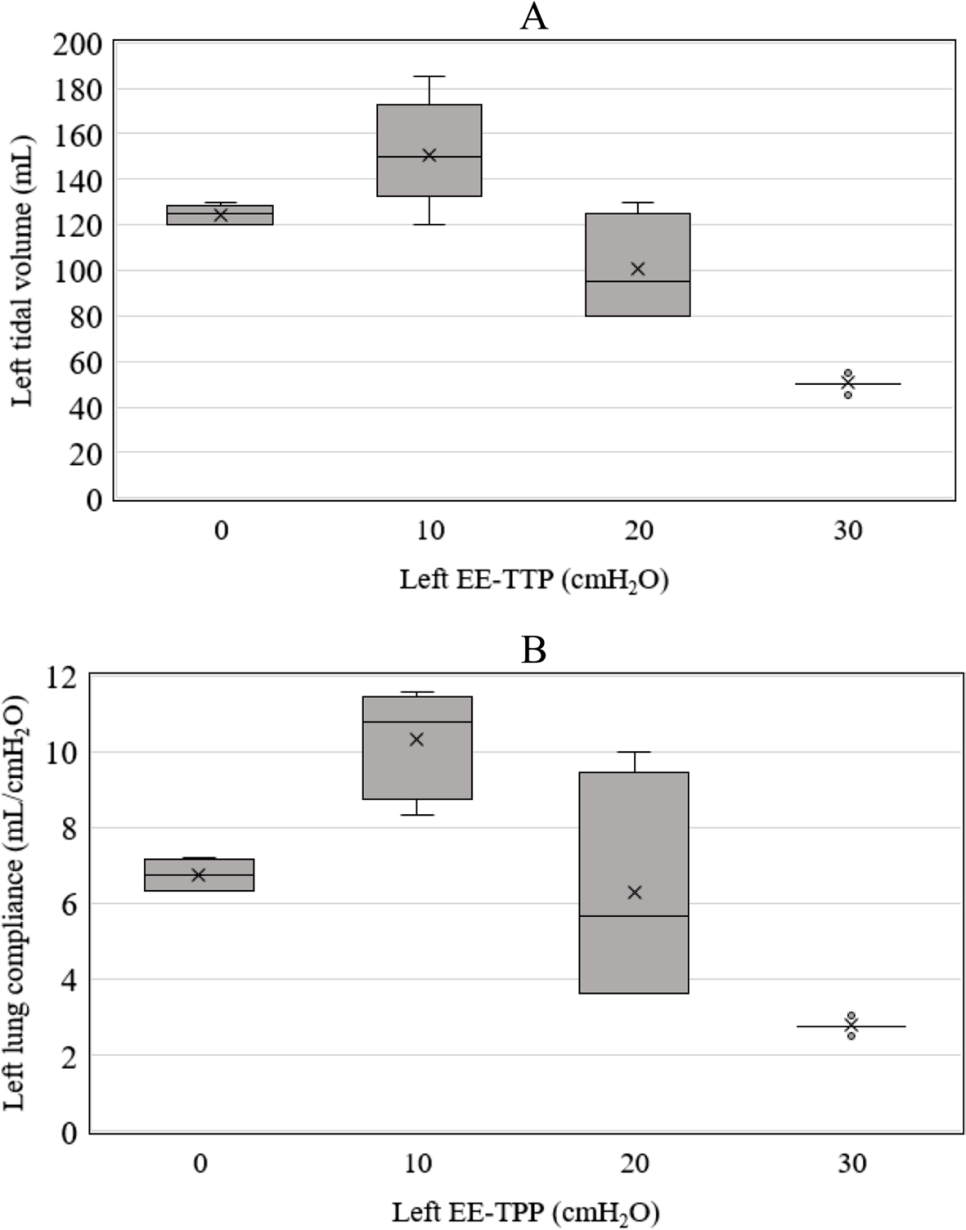
The relationship between the left EE-TPP and left tidal volume is shown in the box-and-whisker plots in **A**. The relationship between the left EE-TPP and left lung compliance is shown in the box-and-whisker plots in **B**. *EE-TPP*, end-expiratory transpulmonary pressure

The relationship between the left EE-TPPs and the LTV is shown in the box-and-whisker plots (Fig. 4A). For better understanding about the above topic, we converted the relationship between the left EE-TPP and the LTV into the left lung compliance as a post hoc analysis. The relationship between the left EE-TPPs and the left lung compliance is shown in the box-and-whisker plots (Fig. 4B). The LTV and the left lung compliance increased as the left EE-TPP increased from 0 to 10 cmH_2_O. On the other hand, as the left EE-TPP increased from 10 to 20 cmH_2_O, or from 20 to 30 cmH_2_O, the LTV and the left lung compliance decreased. When the left EE-TPP was 20 cmH_2_O, there was a large variation in the left lung compliance. This may be because the left EE-TPP consisted of PEEP and chest tube negative pressure, and the left lung compliance differed depending on EE-TPP configuration. The reason why the large difference in left lung compliance was observed between EE-TPP configuration patterns especially at 20 cmH_2_O of EE-TPP could not be clarified in this study, but it is consistent with our hypothesis that the negative pressure from the chest tube changes the transpulmonary pressure, which affects lung compliance. We believe that the LTV and the lung compliance increased when the EE-TPP increased from 0 to 10 cmH_2_O and beyond the LIP, but these decreased when the EE-TPP increased from 10 to 20 cmH_2_O or from 20 to 30 cmH_2_O and beyond the UIP.

It has been reported that the same test lung that we used in the present study may have the LIP and the UIP, and that the compliance of the test lung was improved by a certain amount of PEEP and decreased by excessive PEEP [4]. It was reported that chest drainage worked just like PEEP because it increased EE-TPP [4]. Based on the theory of EE-TPP, the left and right lungs had different EE-TPP when unilateral chest drainage was applied. Therefore, lower chest drainage or PEEP helped to improve lung compliance by appropriately shifting the starting points to around the LIP on the P–V curve. However, higher chest drainage or even lower chest drainage in combination with PEEP worsened lung compliance by shifting the starting points to around the UIP. In addition, higher chest drainage combined with PEEP caused further deterioration of lung compliance by moving the starting points above the UIP, reflecting the drastic decrease in the LTVs. On this point, we observed an interesting finding in this study. Applying PEEP apparently increased the Ppls; however, these increases were inferior to the applied PEEP. This phenomenon was observed even when higher chest drainage was applied in combination with PEEP, which caused further decrease in the left lung compliance. This phenomenon could be explained by the following; total lung compliance improved with applied PEEP compared to no PEEP because driving pressures decreased to achieve the same TVs.

It has been reported that additional chest drainage of 15 cmH_2_O decreased the driving pressure of spontaneous breathing after thoracic surgery [6], while, to the best of our knowledge, there has been no report on chest drainage under mandatory mechanical ventilation. In the present study, chest drainage was assumed under mandatory mechanical ventilation without spontaneous breathing. Apart from the issue of PEEP, in this study, the driving pressure was reduced to ensure 300 mL of TTV in certain situations (by changing the chest drainage from 0 to 10 cmH_2_O in settings 1 and 3), which also suggests an improvement in lung compliance. As mentioned above, shifting the starting point of the left lung P–V curve probably contributed to this phenomenon. The reason why chest drainage decreased driving pressure during spontaneous breathing in the previous report was possibly because the same respiratory mechanism was at work. However, special care should be taken in this regard. Under mandatory mechanical ventilation without spontaneous breathing, the Ppl is equal to the TPP. However, even in such a situation, our results suggest that the TPPs generated by chest drainage were higher than the measured Ppls displayed on the barometer built into the anesthesia machine. In other words, the actual amount of stress on the lung parenchyma may be higher than the values displayed on the anesthesia machine. For example, when chest drainage of 20 cmH_2_O is performed, the peak-TPP may be 20 cmH_2_O higher than the Ppl displayed on an anesthesia machine. This peak-TPP may represent an unacceptable load on the lung parenchyma. More careful control with an understanding of respiratory physiology may be required for mechanical ventilation management during chest drainage.

This study has some limitations. First, it was an experimental study using an anesthesia ventilator and handmade lung-thoracic cavity model. In addition, there may be significant differences in lung compliance, the LIP and the UIP between the test lungs used in this study and real human lungs. Therefore, it is difficult to apply the results of the present study to the actual clinical setting as it is. However, the results of our study need to be considered in actual clinical practice because the improvement in lung compliance or lung overdistention may well occur in real ventilated lungs as they do in the model used in the present study. Second, measurement errors were likely because different respirometers were used to measure the RTV and the LTV. This difference in respirometers was unavoidable due to a lack of resources. However, since the left and right respirometers were not exchanged during the experiment, the RTV and LTV trends are considered reliable.

## Conclusions

The present study demonstrated that unilateral chest drainage may cause unbalanced ventilation of the left and right lungs regardless of PCV or VCV. Mechanical ventilation management with unilateral chest drainage requires careful management because unilateral chest drainage may cause improvement or worsening of ipsilateral lung compliance.

## Data Availability

Data sharing is not applicable to this article, as no datasets were generated or analyzed for the report.

## Abbreviations

TPP: Transpulmonary pressure
PCV: Pressure-controlled ventilation
LTV: Left tidal volume
RTV: Right tidal volume
Ppl: Plateau pressure
TTV: Total tidal volume
PEEP: Positive end-expiratory pressure
VCV: Volume-controlled ventilation
SD: Standard deviation
EE-TPP: End-expiratory transpulmonary pressure
Peak-TPP: Peak transpulmonary pressure
LIP: Lower inflection point
UIP: Upper inflection point
